# Superresolution STED imaging reveals a periodic punctate pattern of adenylyl cyclase type III on primary cilia

**DOI:** 10.1186/2046-2530-1-S1-P32

**Published:** 2012-11-16

**Authors:** J Liao, T Yang, P Hampilos

**Affiliations:** 1 Columbia University, NY, USA

## 

Adenylyl cyclases type III (ACIII) is a primary cilia marker involved in cAMP signaling, playing important roles in regulating ciliogenesis and sensory function. Despite its importance, detailed ACIII localization and their interactions with other proteins remain unclear due to the limited resolution of conventional microscopy. To determine the morphological characteristics of ACIII in primary cilia, we conducted superresolution imaging of immunostained ACIII in fibroblasts and neurons using stimulated emission depletion (STED) microscopy, which allows us to resolve the localization of ACIII achieving a resolution of 50 nm. In contrast to the previous understanding that ACIII distributes uniformly along a primary cilium, our STED images revealed that ACIII formed a periodic punctate pattern with a roughly equal spacing between groups of puncta. These puncta occupied less than 50% of the area, with the size of 137±20 nm in the axial direction along the primary cilia. The spacing between puncta was 250±67 nm. Some primary cilia even showed two rows of periodic puncta along the axial direction, with a tilted angle of about 12° to 35° between the two rows. The spacing between the two rows was 195±19 nm. In some cells, ACIII was only localized in the basal body, where the periodic punctate pattern was absent. In summary, based on our superresolution studies, we found that ACIII can be transported into a primary cilium, but would only occupy regions approximately equally spaced along the cilium.

